# IgG‐Secreting Lymphoplasmacytic Lymphoma Presenting With Massive Kidney Infiltration

**DOI:** 10.1002/ccr3.71290

**Published:** 2025-10-30

**Authors:** Yuichi Nakamura, Yoshihiro Itoh, Tomoyuki Sakamoto, Emi Kakegawa, Yasuhito Terui, Taichi Tarusawa, Tsutomu Inoue, Hirokazu Okada, Keisuke Ishizawa, Taketo Yamada

**Affiliations:** ^1^ Department of Hematology Saitama Medical University Hospital Iruma‐gun Saitama Japan; ^2^ Department of Nephrology Saitama Medical University Hospital Iruma‐gun Saitama Japan; ^3^ Department of Pathology Saitama Medical University Iruma‐gun Saitama Japan

**Keywords:** acute kidney injury, IgG‐secreting, kidney involvement, lymphoplasmacytic lymphoma, non‐IgM

## Abstract

Various factors related to lymphoplasmacytic lymphoma (LPL) can lead to kidney complications; however, LPL‐related kidney complications are not as well‐described as those with multiple myeloma. Here, we report a case of IgG‐secreting LPL presenting with acute kidney injury owing to direct tumor infiltration. Chemotherapy resulted in hematological and renal improvements.

## 1 Introduction

Lymphoplasmacytic lymphoma (LPL) is a neoplasm of the small B lymphocytes, plasmacytoid B lymphocytes, and plasma cells, usually involving the bone marrow (BM) and sometimes the lymph nodes (LNs) and spleen. It is characterized by the *MYD88* L265P mutation, a hallmark diver gene mutation observed in the vast majority of disease [1]. Waldenström macroglobulinemia (WM) is defined as LPL with BM involvement and immunoglobulin (Ig) M monoclonal gammopathy. WM is found in a substantial subset of LPL, bur is not synonymous with it. Nevertheless, cases of LPL without IgM paraprotein secretion (non‐IgM LPL) are uncommon and have not been frequently reported [2, 3].

In lymphoproliferative disorders, kidney diseases can be induced by various mechanisms, including urinary tract obstruction by the LNs, renal parenchymal invasion with tumor cells, and monoclonal Ig‐related nephrotoxicity, such as light chain tubulopathy and amyloidosis. In addition to these direct or indirect consequences of tumor expansion, therapy‐related complications such as tumor lysis, toxicity of drugs, and infections can result in acute kidney injury (AKI).

Although both LPL/WM and multiple myeloma (MM) are hematological conditions that produce a monoclonal gammopathy, renal complications are less common and not well‐described in LPL/WM as compared to MM.

Herein we report a case of non‐IgM LPL presenting with AKI owing to prominent renal parenchymal infiltration with tumor cells.

## 2 Case History/Examination

A 64‐year‐old male patient with no relevant medical history was referred to our hospital with anemia and monoclonal gammopathy. On physical examination, his conjunctivae were pale, but not icteric. No cervical, axillary or inguinal lymphadenopathy, and no organomegaly in the abdomen were observed. Skeletal radiographical examination revealed no apparent destructive bone lesions.

**FIGURE 1:**
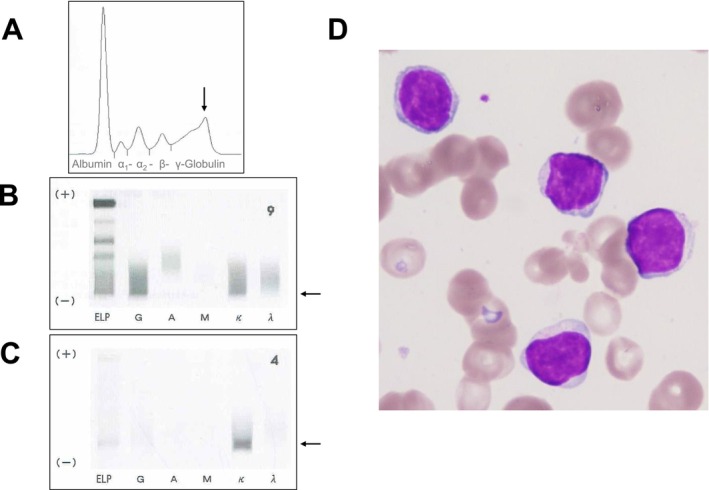
(A) Serum protein electrophoresis presenting M‐spike (indicated by arrow) in *γ* zone. (B) Serum immunofixation presenting IgG‐κ paraprotein (indicated by arrow). (C) Urine immunofixation presenting κ‐type Bence Jones protein (indicated by arrow). (D) Morphologic feature of atypical lymphoid cells in bone marrow (May‐Giemsa staining, original magnification ×1000).

**FIGURE 2:**
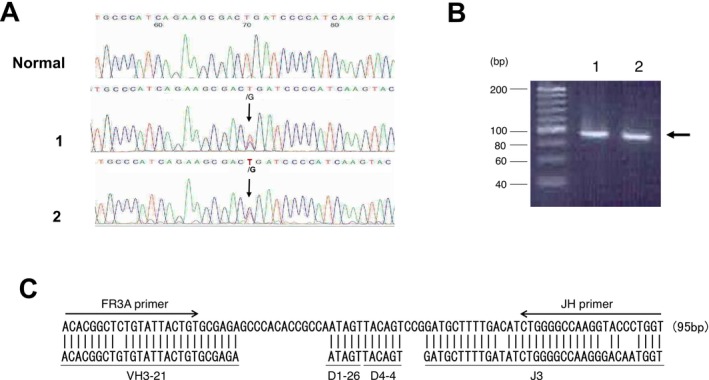
Molecular analysis. (A) Sequence analysis showing *MYD88* L265P mutation. DNA was amplified using primers, 5′‐GGGATATGCTGAACTAAGTTGCCAC‐3′ (forward) and 5′‐GACGTGTCTGTGAAGTTGGCATCTC‐3′ (reverse). PCR product containing the reported mutation point was directly sequenced. Arrows indicated T>G substitution. 1; Bone marrow 2; Renal biopsy samples. (B) Semi‐nested PCR to amplify the complementary‐determining region 3 (CDR3) sequences. Primers were directed the joining region (first round primer, LJH: 5′‐TGAGGAGACGGTGACC‐3′; nested second‐round primer, VLJH: 5′‐GTGACCAGGGTNCCTTGGCCCCAG‐3′) and to the conserved framework 3 (FR3) segment of the variable region (5′‐ACACGGC(C/T)(G/C)TGTATTACTGT‐3′). Electrophoresis of the second PCR products from the two lymphomatous lesions presented the comigrating monoclonal bands (horizontal arrows). Lane 1; Bone marrow Lane 2; Renal biopsy samples. (C) Nucleotide sequences of CDR3 of the samples. PCR products were cloned into plasmid vector and sequenced. The identical sequence derived from the both samples was presented. The primer sequences (FR3A and VLJH) and diversity region (D) were shown by underlines.

Laboratory findings are shown in Table 1 and Figure 1. Peripheral blood showed a hemoglobin level of 7.7 g/dL, white blood cell count of 6.58 × 10^3^/μL with no abnormal lymphocytes, and platelet count of 577 × 10^3^/μL. Serum creatinine level and estimated glomerular filtration rate were normal at that time. Urinalysis revealed microscopic hematuria and was negative for albuminuria. Serum protein electrophoresis showed a small M‐spike coexisting with polyclonal Ig in *γ* zone (Figure 1A), which was confirmed as IgG‐κ paraprotein on immunofixation (Figure 1B). In addition, urine immunofixation revealed κ‐type Bence Jones protein. (Figure 1C). Serum Ig free light chain κ/λ ratio was elevated to 2.5. The BM aspirate showed an increase in lymphocytes (73%), including atypical cells with abundant cytoplasm (Figure 1D); however, no proliferation of plasma cells was observed. Flow cytometry analysis revealed that these lymphocytes expressed CD19, CD20, and cytoplasmic Ig‐κ, but not CD138 or cytoplasmic Ig‐λ. A BM trephine biopsy revealed prominent infiltration of CD20‐positive atypical cells, which were also positive for CD79a and cytoplasmic Ig‐κ and negative for CD3, CD5, CD23, cytoplasmic Ig‐λ, and cyclin D1. Chromosomal analysis of BM aspirate showed normal karyotype. DNA sequence analysis of BM cells revealed the *MYD88* L265P mutation (Figure 2A). Therefore, the patient was diagnosed with LPL (non‐IgM type) and treated with oral administration of cyclophosphamide and prednisolone, resulting in a partial improvement of anemia.

**TABLE 1:** Peripheral blood and chemistry findings during the course of the disease.

	At the diagnosis of LPL	At the start of hemodialysis	At the end of hemodialysis
Urine			
Protein	—	2+	—
Occult blood	2+	3+	—
Sediment			
RBC (/HPF)	10–19	> 100	1–4
WBC (/HPF)	—	1–4	1–4
Granular cast (/ HPF)	—	20–29	—
Peripheral blood			
WBC (×10^9^/L)	6.58	4.82	7.73
Hemoglobin (g/L)	7.7	4.0	10.9
Platelet (×10^9^/L)	577	169	167
Blood chemistry			
Total protein (g/dL)	7.6	6.9	6.8
Albumin (g/dL)	2.6	1.9	4.1
LD (U/L)	186	269	226
Creatinine (mg/dL)	0.59	12.43	3.01
eGFR (mL/min/1.73 m^2^)	105.2	3.7	17.5
BUN (mg/dL)	12.7	74.9	37.0
Uric acid (mg/dL)	5.4	11.3	5.3
Calcium (mg/dL)	8.1	7.3	9.0
CRP (mg/dL)	5.83	10.02	0.18
IgG (mg/dL)	2744	1323	1304
IgA (mg/dL)	318	132	< 10
IgM (mg/dL)	45	14	< 5
FLC‐κ (mg/L)	152.0	1130.0	271.0
FLC‐λ (mg/L)	60.7	190.0	79.5
κ/λ	2.50	5.95	3.41
IL‐2R (U/mL)	3477	18,935	4532

Twelve months after the initial diagnosis, the patient developed diarrhea, nausea, and shortness of breath. Blood examination showed prominent elevation of serum creatinine (12.43 mg/dL) and progression of anemia (Hb 4.0 g/dL) (Table 1). Urinalysis revealed albuminuria and hematuria. Urine sediment included many granular casts and erythrocytes. Serum IgG level was decreased, whereas Ig free light chain κ/λ ratio increased to 5.95. The C3 and C4 levels were 78 mg/dL (normal range: 90–180 mg/dL) and 28 mg/dL (normal range: 10–40 mg/dL), respectively. Cryoglobulin tests were negative and anti‐nuclear antibody, anti‐neutrophil antibody, and anti‐glomerular basement membrane antibody were absent. Computed tomography revealed that he had a horse‐shoe kidney without apparent enlargement. His left renal pelvis was mildly dilated; however, no urinary tract obstruction was detected.

## 3 Differential Diagnosis

The patient was admitted for AKI management and was immediately treated with hydration and red blood cell transfusion, which failed to achieve any improvement. Subsequently, hemodialysis therapy was initiated for treatment of severe AKI.

**FIGURE 3:**
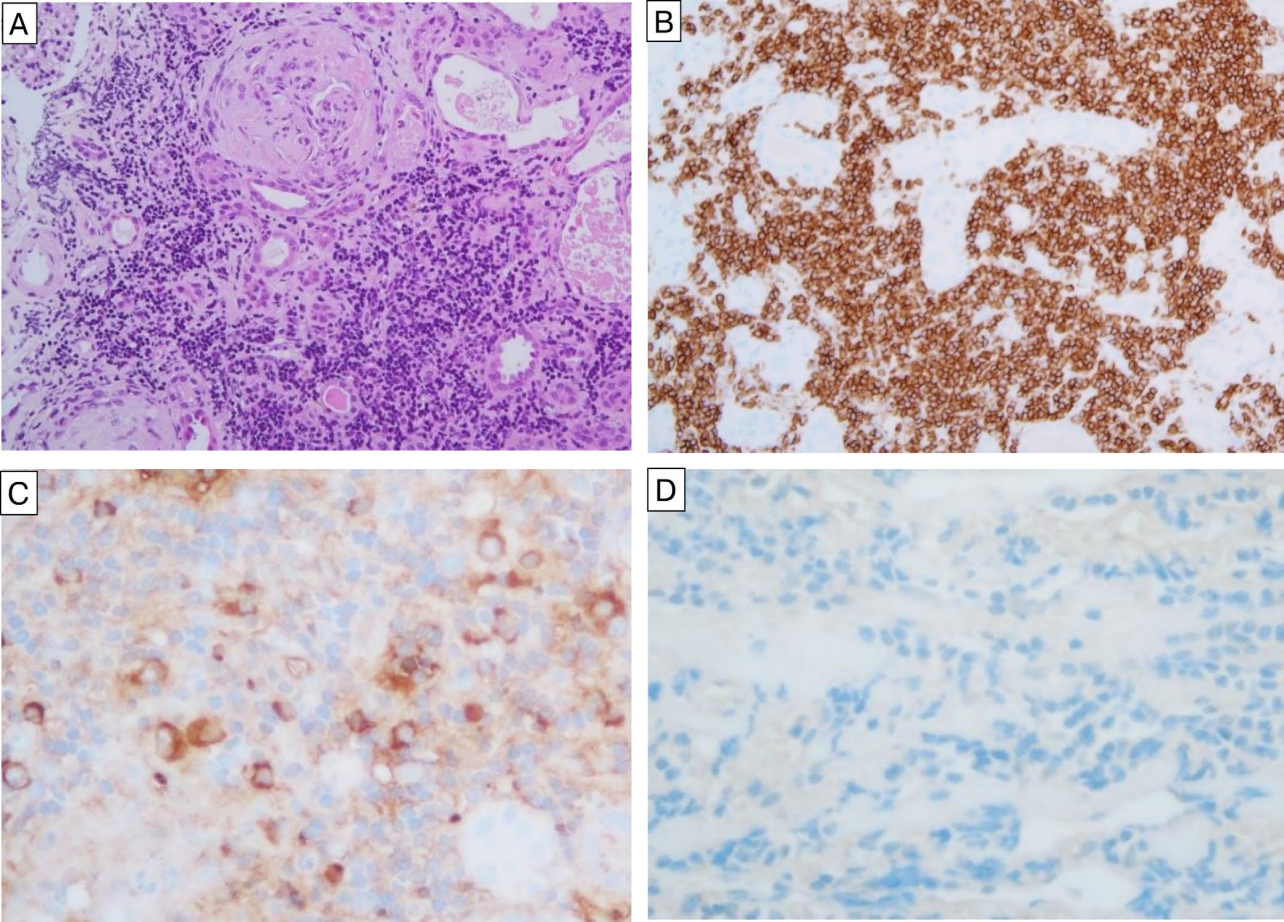
Histopathologic presentation of the renal biopsy. Hematoxylin and eosin (HE) staining (A) and immunostaining with antibodies against CD20 (B) (original magnification ×100), Igκ (C) and Igλ (D) light chain of renal biopsy (original magnification ×200).

An open renal biopsy was performed to determine the cause of renal failure, which revealed a dense and patchy interstitial invasion of atypical lymphoid cells with tubular atrophy and dilatation as well as glomerular crescentic formation (cellular and fibrous) (Figure 3A). Immunohistochemical staining revealed that the infiltrating cells were positive for CD20 (Figure 3B), CD79a, and cytoplasmic Ig‐κ (Figure 3C), and negative for CD3 and cytoplasmic Ig‐λ (Figure 3D). Immunofluorescence staining revealed that the glomeruli were partially positive for IgG, IgA, IgM, C1q, C3c, C4c, and fibrinogen. These findings were nonspecific, thus contributing little to the pathological diagnosis.

To confirm that the atypical cells infiltrating the kidney were of the same origin as the BM lymphoma cells, DNA was prepared from paraffin‐embedded samples and subjected to polymerase chain reaction (PCR) and sequence analysis. As shown in Figure 2A, the *MYD88* L265P mutation was also detected in the renal lesion. In addition, the BM and renal lesions shared an identical rearrangement within the *Ig heavy chain* (*IgH*) gene, as confirmed by sequence analysis (Figure 2B,C). Congo red staining did not reveal amyloid lesions, and no tubular casts were observed.

## 4 Conclusion and Results

Based on the pathological findings of the renal biopsy, it was suggested that the severe renal injury was caused mainly by interstitial lymphomatous infiltration of the kidney. The patient was treated with R‐CHOP (rituximab, cyclophosphamide, doxorubicin, vincristine, and prednisolone) chemotherapy. After four cycles of R‐CHOP, rituximab monotherapy was initiated and continued. Initially the kidney injury seemed to be irreversible; however, with continuous rituximab therapy, the renal function slowly recovered resulting in the weaning of hemodialysis therapy with a serum creatinine level of 3.01 mg/dL 30 months after the start of chemotherapy. Anemia also improved and the serum Ig free light chain κ/λ ratio decreased (Table 1), whereas IgG‐κ paraprotein was still present. The use of Bruton's tyrosine kinase inhibitors is currently under consideration.

## 5 Discussion

Herein we present the case of a patient with non‐IgM LPL, who developed severe AKI owning to the direct renal parenchymal infiltration with tumor cells. In this case, MM was excluded based on the lack of proliferation of plasma cells and bone‐destructive lesions as well as the presence of *MYD88* L265P mutation. Small lymphocytic lymphoma was ruled out because of the negativity for CD5 and CD23, although the atypical cells did not show distinct plasmacytic differentiation. Marginal zone lymphoma was also ruled out as lymphoma cells infiltrated predominantly into BM lacking apparent involvement of lymph nodes, mucosa‐associated lymphoid tissues or spleen. Non‐IgM LPL is a rare entity and previous studies have indicated its heterogeneity and differences from classical WM [2, 3].

Kidney manifestations in LPL/WM are less common than those in MM and the occurrence of renal insufficiency was reported in approximately 3%–8% of WM cases [4, 5, 6, 7, 8]. Although the vast majority of kidney involvement in MM has been attributed to cast nephropathy [9], the disease spectrum in LPL/WM is different. Kidney diseases in WM have been shown to be most likely related to abnormal monoclonal proteins and/or direct tissue infiltration with the neoplasm and are divided into amyloid‐related glomerulopathy, non‐amyloidotic glomerulopathy (cryoglobulinemic or non‐cryoglobulinemic membranoproliferative glomerulopathy, light and heavy chain deposition disease) and tubulointerstitial disease (light chain cast nephropathy, tumor infiltration) [5, 6, 7, 8]. Amyloid light chain (AL) amyloidosis, cryoglobulinemic glomerulopathy, and lymphoma infiltration are common manifestations of LPL/WM and IgM‐secreting B cell lymphoproliferative disorders [5, 6, 7, 8].

The present case with IgG‐secreting LPL developed severe AKI during the course of the disease. The renal biopsy revealed massive interstitial infiltration with atypical cells which were confirmed to be *MYD88*‐mutated B lymphoma cells. No monoclonal gammopathy‐associated amyloidosis, cryoglobulinemic nephropathy, or cast nephropathy were observed. Glomerulopathy with crescentic formation was observed in the biopsied specimens; however, the exact pathophysiology could not be determined. Glomerular crescents can be accompanied by a strong periglomerular inflammatory response, potentially triggered by proinflammatory mediators released from the activated parietal epithelial cells across the Bowman's capsule [10]. Since no intraglomerular tumor infiltration was not evident, we postulated that inflammatory stimulation, such as cytokine release by interstitial lymphoma cells, might activate the parietal cells, resulting in crescentic formation.

Non‐IgM LPL cases presenting with lymphomatous kidney infiltration are very rare and only one case of IgG‐type was reported in the literature [11]. To the best of our knowledge, the present case is the second reporting lymphomatous kidney infiltration in a patient with IgG‐secreting non‐IgM LPL. The previously reported case was a 65‐year‐old man presenting with weight loss, anemia (hemoglobin, 7.0 g/dL), and renal dysfunction (serum creatinine, 1.83 mg/dL). He was initially suspected to have MM, because of the presence of IgG‐κ type monoclonal gammopathy (serum IgG 3825 mg/dL free κ light chain 855.5 mg/L, free λ light chain 97.5 mg/L and κ/λ ratio 97.5) [11]. However, pathological examination revealed renal interstitial infiltration and BM proliferation of CD20‐ and CD138‐positive lymphoid cells, which led to a diagnosis of LPL. The patient was successfully treated with corticosteroid and chemotherapy. In this previous case, renal impairment was relatively mild compared to that in our case, despite a similar pattern of kidney invasion. Dehydration owning to diarrhea and nausea may have resulted in the rapid development of AKI in the present case.

The differential diagnosis of MM is very important, especially in a setting of renal impairment accompanied by non‐IgM type monoclonal gammopathy. When LPL is diagnosed, renal biopsy should be considered to determine the cause of renal injury, including various LPL‐unrelated underlying morbidities.

Our patient exhibited a hematological response to anti‐lymphoma chemotherapy (R‐CHOP); however, the severe kidney injury initially seemed irreversible. After continuous rituximab therapy, the patient showed a slow renal response and was finally weaned off hemodialysis. A previous report indicated that a good renal response was associated with hematological effectiveness [4].

Therefore, physicians should be aware of possible kidney manifestations in patients with LPL/WM in order to provide appropriate treatment based on the prompt and accurate evaluations.

## Author Contributions

**Yuichi Nakamura:** conceptualization, investigation, methodology, project administration, supervision, visualization, writing – original draft, writing – review and editing. **Yoshihiro Itoh:** conceptualization, resources, writing – original draft. **Tomoyuki Sakamoto:** data curation, writing – review and editing. **Emi Kakegawa:** data curation, investigation, writing – review and editing. **Yasuhito Terui:** conceptualization, writing – review and editing. **Taichi Tarusawa:** resources, writing – review and editing. **Tsutomu Inoue:** resources, writing – review and editing. **Hirokazu Okada:** resources, writing – review and editing. **Keisuke Ishizawa:** investigation, resources, writing – review and editing. **Taketo Yamada:** investigation, resources, writing – review and editing.

## Consent

Written informed consent was obtained from the patient for publication.

## Conflicts of Interest

The authors declare no conflicts of interest.

## Data Availability

The data that support the findings of this study are available on request from the corresponding author. The data are not publicly available due to privacy or ethical restrictions.
